# Numerical Assessment on Rotation Effect of the Stagnation Surface on Nanoparticle Deposition in Flame Synthesis

**DOI:** 10.3390/ma12091361

**Published:** 2019-04-26

**Authors:** Lilin Hu, Zhu Miao, Yang Zhang, Hai Zhang, Hairui Yang

**Affiliations:** Key Laboratory for Thermal Science and Power Engineering of Ministry of Education, Tsinghua University-University of Waterloo Joint Research Center for Micro/Nano Energy & Environment Technology Department of Energy and Power Engineering, Tsinghua University, Beijing 100084, China; hll17@mails.tsinghua.edu.cn (L.H.); oceanskymz@126.com (Z.M.); yang-zhang@tsinghua.edu.cn (Y.Z.); yhr@tsinghua.edu.cn (H.Y.)

**Keywords:** flame synthesis, flame stabilizing on a rotating surface (FSRS), rotational speed, particle deposition, Karlovitz number

## Abstract

The effect of rotation of the stagnation surface on the nanoparticle deposition in the flame stabilizing on a rotating surface (FSRS) configuration was numerically assessed using CFD method. The deposition properties including particle trajectories, deposition time, temperature and surrounding O_2_ concentration between the flame and stagnation surface were examined. The results revealed that although flame position is insensitive to the surface rotation, the temperature and velocity fields are remarkably affected, and the deposition properties become asymmetric along the burner centerline when the surface rotates at a fast speed (rotational speed *ω* ≥ 300 rpm). Particles moving on the windward side have similar deposition properties when the surface rotates slowly, but the off-center particles on the leeward side have remarkable longer deposition time, lower deposition temperature, and lower surrounding O_2_ concentration, and they even never deposit on the surface when the surface rotates at a high speed. The rotation effect of the stagnation surface can be quantitatively described by an analogous Karlovitz number (*Ka’*), which is defined as the ratio of characteristic residence time of moving surface to the aerodynamics time induced by flame stretch. For high quality semiconducting metal oxide (SMO) films, it is suggested that *Ka’* ≥ 1 should be kept.

## 1. Introduction

Nano-sized semiconducting metal oxide (SMO) materials such as TiO_2_, SnO_2_, and ZnO are widely used in photocatalysis, gas sensors and solar cells [1,2,3]. A few techniques have been developed to fabricate nanoparticles, such as the sol-gel method [4], co-precipitation method [5], hydrothermal method [6], impregnation method [7,8], colloidal method [9], and flame synthesis method [10,11]. Among them, the flame synthesis method has a great potential for massive production due to its merits of high throughput, simple post treatment and relatively low cost [12,13]. During the synthesis, the precursors doped in the fuel-oxidizer mixtures undergo rapid decomposition and oxidation in a high temperature flame zone, and the vapor-phase metal oxides turn into fine particles through nucleation, collision and sintering in the post flame zone [14,15]. Clearly, the temperature and velocity distributions in the post flame zone are of great significance for nanoparticle size, uniformity, and deposition on the film.

To well control the thickness and quality of SMO films in a single-step gas-to-film deposition process, flame stabilizing on a rotating surface (FSRS) method was proposed by Wang et al. [16]. This method uses an aerodynamic nozzle to generate a laminar premixed flat flame opposing to a film substrate. The substrate is affixed with a rotating disk which is cooled by the ambient air or cooling water [17,18]. Due to the large temperature gradient between the flame sheet and the cold solid surface, a strong thermophoretic force is induced, driving synthesized particles to deposit on the substrate to form a SMO film within a few milliseconds. Previous studies [16,17,19,20,21] found that FSRS method can well control flame temperature, particle deposition time and gas composition, so it is effective to obtain the desired crystal phase of the nanoparticles, and fabricate sensing films with high sensitivity, selectivity, and stability performance.

However, the previous studies are mostly done with a specific or a narrow range of the rotational speed of the stagnation surface. The rotation effect of the stagnation surface or the film substrate is scarcely assessed. In some studies [16,19], flame position and shape were even assumed to be barely affected by the rotating stagnation surface. In fact, it is straightforward that when the stagnation surface rotates very fast, the ambient cold gas could be entrained into the space between the flame sheet and the stagnation surface. If so, the particle deposition time, local temperature and O_2_ concentration could be affected, resulting in the different size and phase of the synthesized particles [16]. To properly set up the operational parameter for FSRS flame synthesis process, it is necessary to assess the influence of the rotational speed.

Therefore, in this paper, 3-D CFD simulations on the stagnation flow with FSRS setting, especially in the post flame zone, are conducted at different rotational speed (*ω*) in a range of 0 to 600 rpm (round per minute). Based on the simulated velocity and temperature fields, the effects of rotational speed of the stagnation surface on deposition process, including the deposition time, temperature and O_2_ concentration are assessed. A guide to select a proper *ω* for the rotating stagnation surface is to be provided. The study is also helpful to understand the effect of rotating surface on the stagnation flames.

## 2. Numerical Methods 

**Figure 1:**
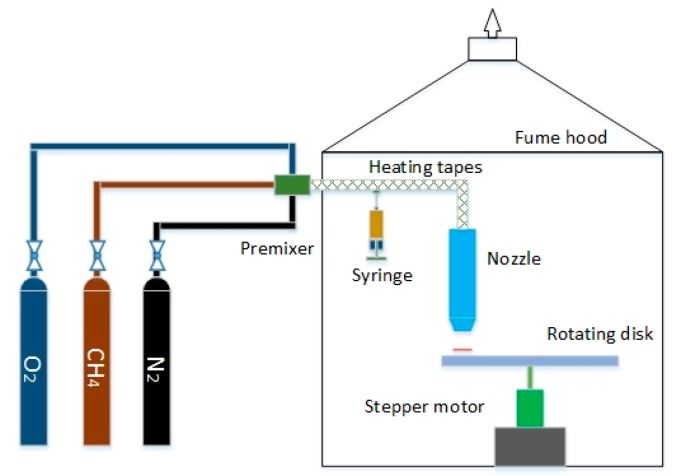
Schematic diagram of the experimental system using flame stabilizing on a rotating surface (FSRS) method [18].

Figure 1 is schematic diagram of the experimental system using FSRS method [18]. A nozzle is placed above a rotating disk. When the combustible mixture is lit, a flame is stabilized between the nozzle exit and the rotating disk, and the top surface of the disk becomes a stagnation surface. The position of the flame depends on the fuel properties and the aerodynamic stretch induced by the imposing flow. For sensor fabrication, a set of substrates are mounted on the solid surface and right below the nozzle exit.

**Figure 2:**
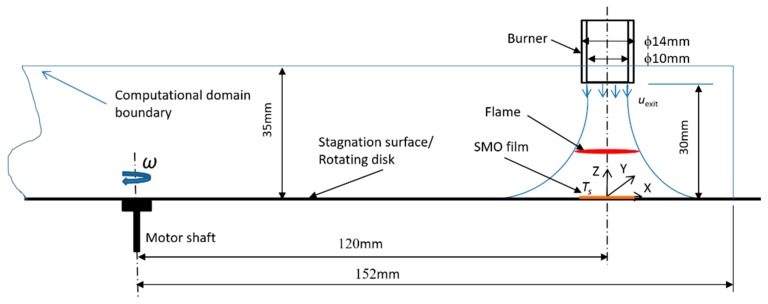
Schematic of the computational domain for the FSRS simulation.

The computational domain is shown in Figure 2. Similar to the configuration used in the previous study [18], the centerline of burner offsets 120 mm from that of the disk, which spins at *ω* rpm. The distance between the burner exit and the stagnation surface is 30 mm. The burner exit has an inner diameter of 10 mm. Inert coflow (Ar) is supplied from the external circular outlet with inner and outer diameters of 11 mm and 14 mm respectively. Namely, the thickness of nozzle at exit is 0.5 mm, and thus the edge of the nozzle exit is assumed to be infinitely thin in CFD simulation. The overall dimension of the computational domain is 304 mm in diameter and 35 mm in height. The organometallic precursors-doped premixed mixtures are injected from the fixed burner, stabilizing a premixed flame above the rotating stagnation surface. Several substrates are placed on the stagnation surface. The horizontal dimension of the substrate is 10 mm × 10 mm.

**Figure 3:**
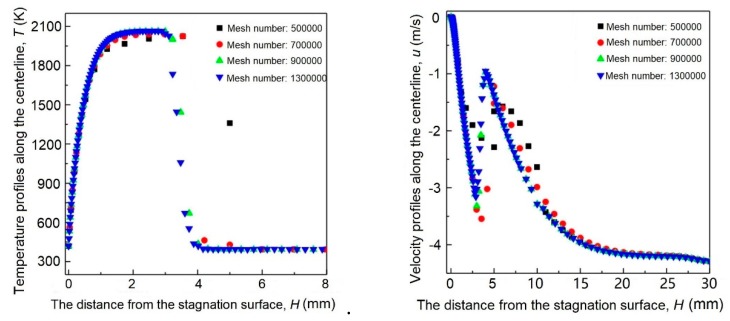
Mesh independence test: (**a**) Temperature profiles along the centerline of the flame at different mesh number; (**b**) velocity profiles along the centerline of the flame at different mesh number.

**Figure 4:**
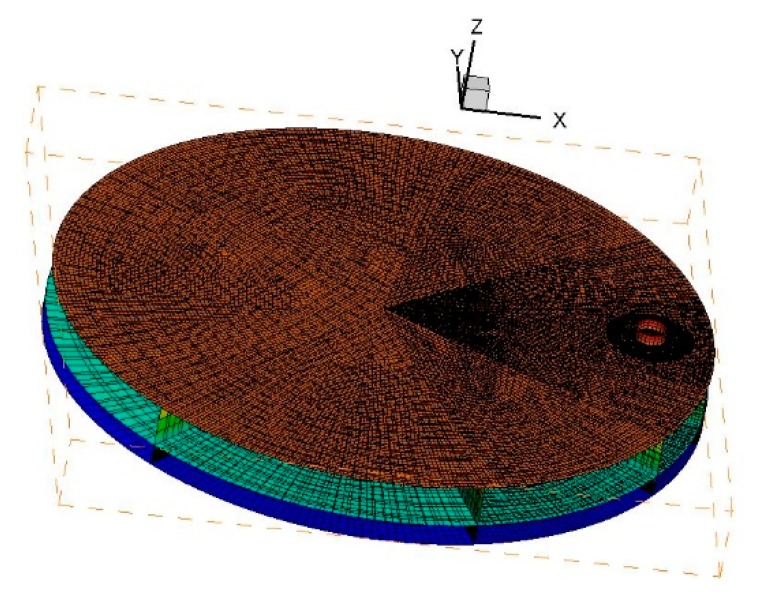
Mesh of the computational domain.

As shown in Figure 2, the *Z* axis denotes the direction along the axis of the burner. The *X* and *Y* axis denote the radial and tangential direction of the rotating disk. In *Z* direction, the bottom region includes the thin gas layer between the rotating surface and the flame, and thus exponential meshing is adopted. The rest region includes the jet flow and surrounding environment, and uniform meshing is employed. In *X*- and *Y*-directions, the domain is divided into nine sectors. Denser gridding is used in the sectors close to the nozzle centerline. The mesh size increases with the distance from the nozzle. Before the simulations, the grid-independency test is performed. The temperature and velocity profiles along the centerline of the flame are obtained when the number of mesh is 500,000, 700,000, 900,000, and 1,300,000, as shown in Figure 3. It can be seen that for mesh number higher than 900,000, the temperature and velocity profiles barely change with mesh number. Therefore, the total mesh number is set to ~1 M. The meshing is presented in Figure 4.

Consistent with the reported experiments [19], simulations are conducted for the lean premixed C_2_H_4_/O_2_/Ar flames (3.9% C_2_H_4_-29.5% O_2_-Ar, equivalence ratio *ϕ* = 0.4) at an initial temperature of 393 K. The velocity at the nozzle exit is 4.29 m/s and 5.52 m/s for the premixed reacting gas and co-flow gas (Ar), respectively. The Ar flow is injected to prevent the impact of surrounding air to the deposition process. Correspondingly, Reynolds number is estimated as 1700 and 2070 and the mean strain rate is 143 s^−1^ and 174 s^−1^.

The temperature of the stagnation surface *T*_s_ (*Z* = 0) will change with *ω*. According to the previous experimental study [16], the surface temperature, *T*_s_ is different at different *ω*’s, and their relationship can be expressed by an empirical correlation *T*_s_ (K) = 464 − 0.15 *ω*, in which the unit of *ω* is rpm (round per minute).

A modified 3-step global mechanism [22] is adopted to describe the chemistry of C_2_H_4_/O_2_/Ar mixtures. The global mechanism is integrated into Fluent in Chemkin format. The thermal and transport parameters of the species are retrieved from USC-Mech II files [23]. Viscous model is selected for laminar simulation, and thermal diffusion is considered. The burner exits are set as velocity boundaries with a constant temperature of 393 K. The other boundaries are set as pressure outlet and the ambient gas is air. The bottom boundary is set as non-slip wall boundary rotating around *Z*-axis. Uniform velocity distribution is set for the exits of the premixed unburned gas and the coflow gas. Steady and pressure-based solver is used.

Particle movements are also studied in the simulation. The central flame section is selected for analyses along the burner centerline. Based on the classical theory, thermophoretic velocity *V*_r_ of particles can be calculated by Equation (1) [24], in which *T* is the local temperature, K; ν is the local gas kinematic viscosity, m^2^/s; and *a* is momentum accommodation coefficient to describe the momentum exchanges during particle collision and is normally set as 0.9 [25].

(1) $$V_{r}=\frac{-3\nu \cdot \nabla T}{\left(4+0.5\pi a\right)T}$$

The particle velocity is the summation of fluid velocity and thermophoretic velocity. With the particle velocity, the particle path from the flame sheet to the stagnation surface is determined.

**Figure 5:**
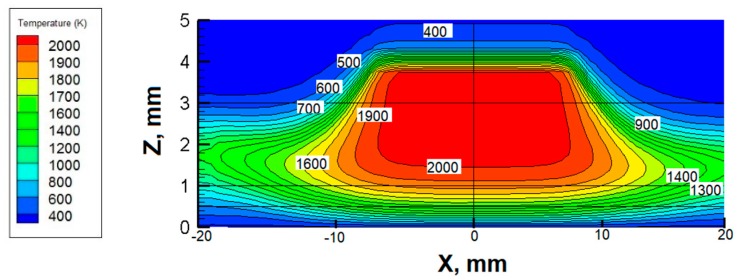
Temperature contours at *ω* = 600 rpm in *X*-*Z* plane (unit: K).

As shown in Figure 5, the temperature distributions on *X*-*Z* plane are symmetric along the nozzle axis even when *ω* = 600 rpm, indicating that the rotational speed has slight influence on *X*-*Z* plane. Given that the rotation of the stagnation surface may have the greatest influence on the tangential direction, the *Y*-*Z* slice (40 mm width × 5mm height) along the flame centerline is selected. The windward side of the flame refers to the area where *Y* ≤ 0 and the leeward side is the area where *Y* > 0 according to the rotation direction of the surface.

## 3. Results and Discussions

### 3.1. Axial Velocity Contours at Different Rotational Speeds

**Figure 6:**
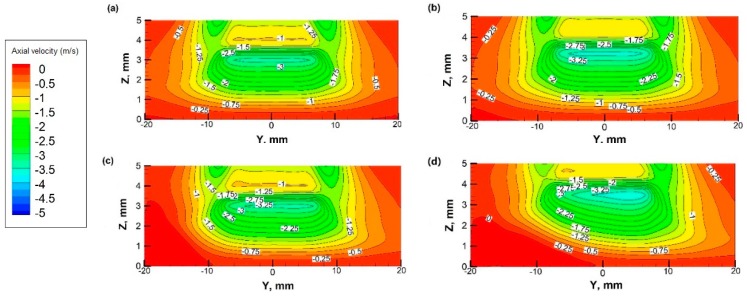
Axial velocity contours at different rotational speed in *Y*-*Z* plane (unit: m/s): (**a**) *ω* = 0; (**b**) *ω* = 100 rpm; (**c**) *ω* = 300 rpm; (**d**) *ω* = 600 rpm.

Figure 6 shows the axial velocity contours adjacent to the flame and the stagnation surface at different *ω*’s. When the disk is in stationary (*ω* = 0), axial velocity distribution is symmetric along the centerline of the nozzle. When the disk rotates (*ω* > 0), the velocity field on the windward side is pushed upwards. The phenomenon is more obvious as *ω* increases. When *ω* = 600 rpm, the influencing zone covers nearly the entire space between the flame and stagnation surface. While *ω* ≤ 300 rpm, the influencing zone is limited to the space near the edge of the flame on the windward side. However, the central area under the flame, i.e., the main synthesis zone is barely affected. Since the horizontal area of the substrate of the SMO film is usually smaller than 10 mm × 10 mm, the influence of the surface rotation is minor when *ω* ≤ 300 rpm, validating the assumption used in the previous study [19].

### 3.2. Temperature Fields at Different Rotational Speeds

**Figure 7:**
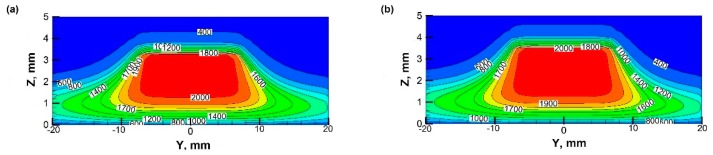

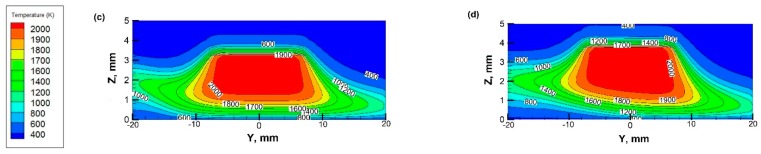
Temperature contours at different rotational speed in *Y*-*Z* plane (unit: K): (**a**) *ω* = 0; (**b**) *ω* = 100 rpm; (**c**) *ω* =300 rpm; (**d**) *ω* = 600 rpm.

Figure 7 shows the temperature contours in the central section adjacent to the stagnation surface at different *ω*’s.

It can be seen that the flame is stabilized ~3 mm above the stagnation surface, with a diameter of ~30 mm. When the surface is in stationary or rotates slowly (e.g., *ω* ≤ 100 rpm), the temperature distribution in the synthesis space is symmetric along the centerline of the nozzle. However, when *ω* = 600 rpm, the temperature fields are very asymmetric, as some cold gas is induced into the bottom of the flame on the windward side. Again, the temperature field results indicate the flame is insignificantly affected by the rotating surface when *ω* ≤ 300 rpm. Based on the temperature and velocity distribution, it is clear that the influence of the rotating stagnation surface is mainly concentrated in the near wall area where the deposition of nanoparticles occurs. While the nucleation and growth of particles near the flame front remain almost unaffected. Therefore, the deposition process is particularly studied.

### 3.3. Particle Deposition Trajectory at Different Rotational Speeds

**Figure 8:**
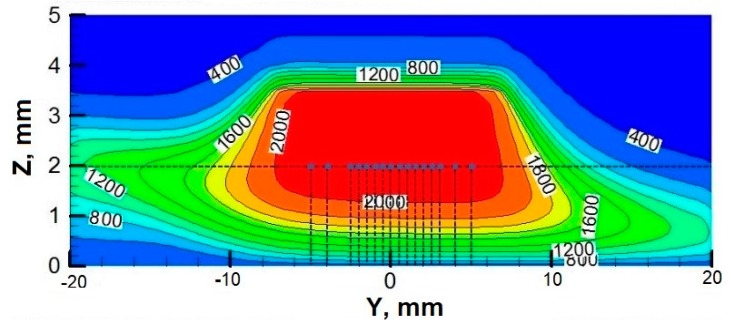
Schematic of the initial settings for tracked particles (The case with *ω* = 300 rpm).

To exam the rotation effect on the deposition process, the deposition trajectories, time, temperature, and mean surrounding O_2_ concentration of 17 selected particles in particle deposition zone are computed at different *ω*’s. The above temperature distributions show that the temperature on the plane 2 mm above the stagnation surface have very small difference at different *ω*’s. Since the horizontal dimension of the substrate of film is about 10 mm × 10 mm, the initial positions of the tracked particles are set on the plane 2 mm above the stagnation surface, shown as the solid dots in Figure 8. The initial radial position of the particles *y*_0_ locates at 0, ±0.5 mm, ±1.0 mm, ±1.5 mm, ±2.0 mm, ±2.5 mm, ±3.0 mm, ±4.0 mm and ±5.0 mm respectively.

**Figure 9:**
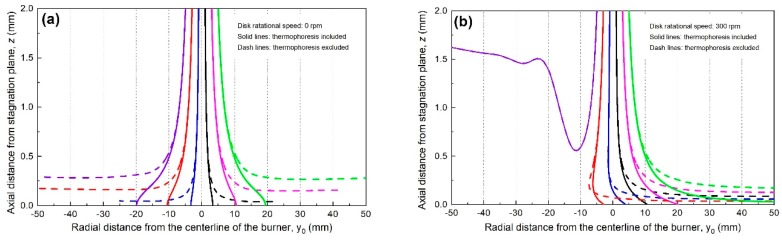
Particle trajectories of the tracked particles when the stagnation surface is (**a**) in stationary and (**b**) *ω* = 300 rpm (at the same surface temperature).

Figure 9 depicts the deposition paths of the tracked particles when *ω* = 0 and *ω* = 300 rpm respectively, both at the same surface temperature (*T_s_* = 419 K). As expected, when *ω* = 0, particle deposition paths are symmetrically distributed along the central axis. Due to the thermophoretic force, the tracked particles deposit within a circle of *ϕ*40 mm on the stagnation surface. When the thermophoretic force is arbitrarily excluded, the tracked particles move with the gas flow and none of them deposits on the film. Under the condition with *ω* = 300 rpm, some particle paths become asymmetric, with the trajectories shifting to leeward side. The tracked particles away from the centerline on the leeward side deposit outside the circle of *ϕ*40 mm. While on the windward side, some particles are entrained into the upward flow generated by thin gas layer of the moving stagnation surface and the flame jet flow. As a result, some particles cannot even approach the wall surface. The closer to the stagnation surface, the greater the impact is. Consequently, particle deposition efficiency decreases.

### 3.4. Particle Deposition Time at Different Rotational Speeds

**Figure 10:**
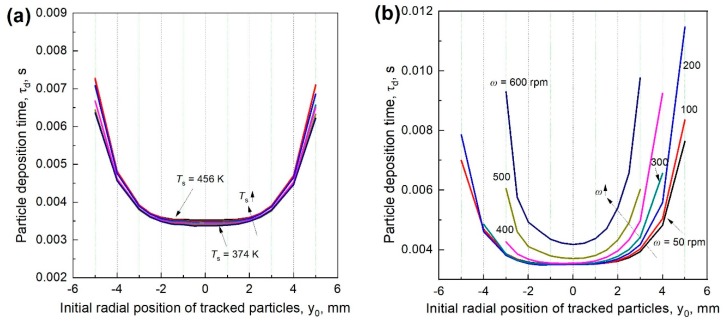
Deposition time of the tracked particles when the stagnation surface is (**a**) in stationary and (**b**) rotates at different speeds.

Deposition time of the tracked particle *τ_d_* is defined as the time that particle experiences from the initial tracked position (near the flame front) to its deposition location on the stagnation surface. Figure 10a shows the variation of *τ_d_* with *T*_s_ when *T*_s_ = 374 − 456 K, corresponding to the *T*_s_’s when surface rotates in the range of 50–600 rpm. Clearly, *τ_d_* is insensitive to *T*_s_. It only decreases a little at high *T*_s_ because of large thermophoretic force and remains nearly constant for particles impinging from the central flame surface (d ≤ 5 mm). Out of the central flame surface, *τ_d_* increases rapidly due to the weak thermophoretic force.

When the surface rotates, shown in Figure 10b, *τ_d_* remarkably changes with *ω*. With the increase of *ω*, *τ_d_* increases and the particles with constant *τ_d_* are limited to a smaller area. This phenomenon can be attributed to the entrained cold gas around the moving stagnation surface, leading to a smaller temperature gradient in the near wall region, thus a weaker thermophoretic force. When *ω* ≤ 300 rpm, the particles right above the solid surface still have the approximate *τ_d_*. For the particles out of the deposition area, *τ_d_* is more sensitive to *ω* on the leeward side. When *ω* > 300 rpm, *τ_d_* increases rapidly with *ω*. For the particles whose initial positions are not in the flame center, the variation trend is more obvious. Consistent with the trajectory results, particles further away from the flame center has infinite *τ_d_*. The results show that when *ω* ≤ 300 rpm, the particles deposit on the stagnation surface have nearly the same *τ_d_*, which is conducive for high-quality and uniform film products.

### 3.5. Particle Deposition Temperature at Different Rotational Speeds

**Figure 11:**
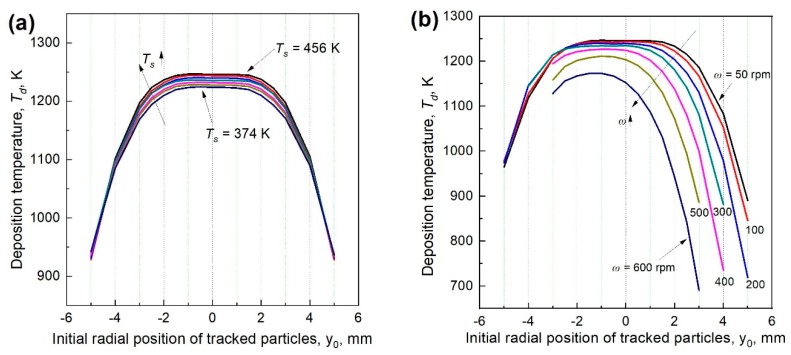
Deposition temperature of the tracked particles when the stagnation surface is (**a**) in stationary and (**b**) rotates at different speeds.

Since the nanoparticles are very small, their temperatures are assumed to be equal to the fluid temperature. Particle deposition temperature, *T_d_*, is the average particle temperature during the deposition process. As shown in Figure 11, *T_d_* is insensitive to *T_s_*. When *ω* = 0, *T_d_* in the central flame region is relatively uniform, while *T_d_* far away from the flame center is much smaller. When *ω* > 0, *T_d_* becomes smaller for all tracked particles as some low temperature ambient gas is entrained into the flame region. At a large *ω*, especially when *ω* > 300 rpm, *T_d_* with different initial positions are of great difference. On the windward side, only the particles around the flame center can deposit on the surface, and they mostly have rather high *T_d_*. However, slightly off the centerline on the leeward side, e.g., when *y*_0_ = 1 mm, *T_d_* remarkably decreases from the peak value, and this is because particles on the leeward side are blown away from the flame center zone and deposit in a low temperature zone as shown in Figure 8. In the center area with *ϕ*20 mm, the effect of the rotating surface on *T_d_* is minor when *ω* ≤ 300 rpm.

### 3.6. Particle Deposition O_2_ Concentration at Different Rotational Speeds

**Figure 12:**
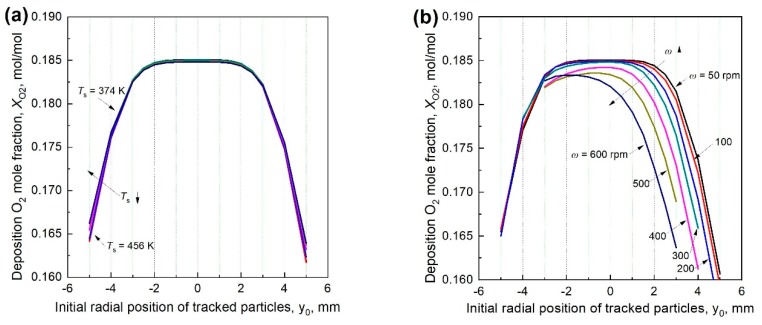
Averaged O_2_ concentration around the tracked particles when the stagnation surface is (**a**) in stationary and (**b**) rotates at different speeds.

The averaged O_2_ mole fraction *X_O2_* in the deposition zone between the flame and surface is important for the product phase [26]. Figure 12 show the *X_O2_* for the tracked particles at different *T_s_*’s varying from 374 K to 456 K. When *ω* = 0, *X_O2_* is insensitive to *T_s_*. For the particles moving down to the surface from flame within the area of *ϕ*5 mm, *X_O2_* is rather constant, while particles from the edge of the flame experience lower *X_O2_*. The further are the particles away from the flame center, the lower *X_O2_* is. When the surface rotates at a slow speed, e.g., *ω* ≤ 300 rpm, *X_O2_* for the tracked particles are approximately the same near the center of the flame, while it decreases remarkably for the particles moving down on the leeward side. When *ω* > 300 rpm, *X_O2_* of all tracked particles decreases with *ω*. The decrease rate is higher when the particles are located further away from the center or at a higher *ω*. In addition, the influence on the leeward particles is more significant than the windward side.

### 3.7. The Effect of the Tangential Velocity and Flame Stretch Rate

**Figure 13:**
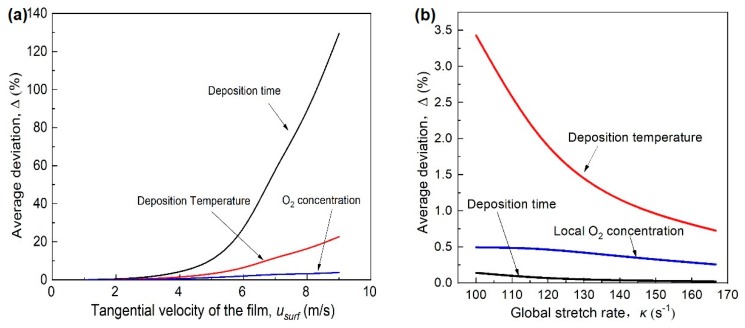
The effects of (**a**) tangential velocity and (**b**) stretch rate on the nanoparticle deposition.

The tangential velocity $u_{surf}\;=\;\omega \cdot r$, in which *r* is the distance of the film to the axis of the disk, is more reasonable to be used to evaluate the impact of rotational stagnation surface on deposition process since it includes the radial position of the film substrate. In Figure 13a, the average deviation Δ represents the difference caused by disk rotation. It can be seen that increasing the tangential velocity (rotational speed) has the most profound influence on the deposition time *τ_d_*, following by the deposition temperature *T_d_* and the local O_2_ concentration.

The flame stretch is a dominant factor to the flow and temperature fields for the stagnation flame. Thus, for FSRS flame synthesis process, the flame stretch should be another key influencing factor besides the rotational speed, or tangential velocity. In the stagnation configuration, the intensity of global flame stretch can be expressed as: ${k\;=\;u}_{exit}\;/\;L$, in which *u_exit_* is the exit velocity of the flow and *L* is the distance between the nozzle and the solid surface.

Figure 13b shows the variation of the deposition performance caused by the flame stretch, based on the simulation results at different *u_exit_*’s with *ω* = 300 rpm. It can be seen the increasing of *k* will reduce the deviation of *τ_d_*, *T_d_* and O_2_ concentration in the deposition zone, opposite to the increasing of tangential velocity. The stretch rate effect is weak on *τ_d_* and O_2_ concentration, but still significant on *T_d_*.

Indeed, the rotation of stagnation surface induces a characteristic residence time in the horizontal direction for the deposition zone, and this characteristic time can be expressed as $\tau_{surf}{\;=\;D}_{f}{\;/u}_{surf}$, where *D_f_* is the diameter of the flame front, approximate to the nozzle diameter. The flame stretching introduces a characteristic residence time in vertical direction. This characteristic time can be expressed as $\tau_{fl}\;=\;1\;/\;k$. Combining the two effects, we can quantify the total effects by introducing an analogous non-dimensional Karlovitz number (*Ka‘*) [27,28], which is defined as the ratio of characteristic residence time induced by the moving surface to the aerodynamic time induced by flame stretch.

(2) $$Ka’\;=\;\frac{\tau_{surf}}{\tau_{fl}}{\;=\;D}_{f}{\;/\;u}_{surf\;}\cdot k$$

**Figure 14:**
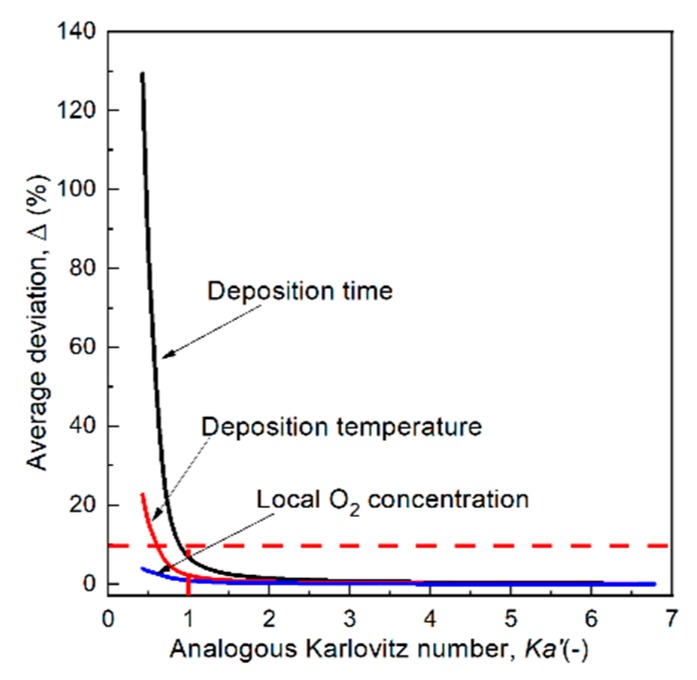
Relation between the average deviation of deposition parameters and analogous Karlovitz number.

Figure 14 shows the variation of the average deviations of *τ_d_*, *T_d_* and O_2_ mole fraction with *Ka’*. It can be seen that the deviations for these deposition properties decrease rapidly with increasing *Ka’*, and becomes less than 10% when *Ka’* ≥ 1. Basically, the deposition O_2_ concentration and *T_d_* are more weakly affected by *Ka‘*, and *τ_d_* is greatly affected by *Ka‘*. For high quality SMO films, it is suggested that *Ka‘* ≥ 1 should be kept.

## 4. Conclusions

FSRS (flame stabilizing on a rotating surface) is a proved method with a single step deposition for nano SMO film fabrication. With well controlled flame temperature, particle deposition time and gas composition, it is effective to obtain the desired crystal phase of the nanoparticles, and fabricate sensing films with high sensitivity, selectivity, and stability performance. However, the temperature and velocity fields for the nano particle deposition could be significantly influenced by the rotation of the stagnation surface. In this paper, 3-D CFD simulation was conducted to assess the effect of rotating surface on the nanoparticles deposition in the FSRS configuration for the premixed C_2_H_4_/air flames. It was found that although flame position is insensitive to the rotation of stagnation surface, the temperature and velocity fields could be remarkably affected. When the surface rotates slowly, the temperature and velocity fields near the flame barely change. When the surface rotates at a fast speed (e.g., *ω* > 300 rpm), the flame on the windward side tilts upward and the entire flame moves to the leeward side. Based on the simulated results, the deposition trajectories, time, temperature, and mean surrounding O_2_ concentration of selected particles between the flame and the surface are computed at different surface rotational speeds. When the surface is in stationary, the deposition of the particles is caused by the thermophoretic force and symmetric along the nozzle centerline. Those deposition properties for the particles moving from the flame center are insensitive to the surface temperature variation, which could be caused by the surface rotation. The deposition properties for the particles from the flame center zone are rather close. When the surface rotates slowly (*ω* ≤ 300 rpm for the present configuration), the particles moving from flame center on the windward side have similar deposition properties. The particles from the flame but in off-center area on the leeward side have remarkable longer deposition time, lower deposition temperature and lower surrounding O_2_ concentration. When the surface rotates faster, the changes in deposition properties are severer. The effect of rotational surface can be described by analogous Karlovitz number (*Ka‘*), which is defined as the ratio of characteristic residence time of moving surface to the aerodynamics time induced by flame stretch. With proper settings of the operational parameters of FSRS method, the negative impacts caused by the rotation of the stagnation surface could be minimized. Based on the simulation results of this paper, for high quality SMO films, it is suggested that *Ka‘* ≥ 1 should be satisfied.
